# Ionic and Amino Acid Regulation in Hard Clam (*Meretrix lusoria*) in Response to Salinity Challenges

**DOI:** 10.3389/fphys.2016.00368

**Published:** 2016-08-25

**Authors:** Chia-Hao Lin, Po-Ling Yeh, Tsung-Han Lee

**Affiliations:** ^1^National Institute for Basic Biology, National Institutes of Natural SciencesOkazaki, Japan; ^2^Department of Life Sciences, National Chung Hsing UniversityTaichung, Taiwan; ^3^Agricultural Biotechnology Center, National Chung Hsing UniversityTaichung, Taiwan

**Keywords:** hard clam, osmoregulation, Na^+^, K^+^-ATPase, taurine transporter, free amino acid

## Abstract

Most marine mollusks are osmoconformers, in that, their body fluid osmolality changes in the direction of the change in environmental salinity. Marine mollusks exhibit a number of osmoregulatory mechanisms to cope with either hypo- or hyperosmotic stress. The effects of changes in salinity on the osmoregulatory mechanisms of the hard clam (*Meretrix lusoria*, an economically important species of marine bivalve for Taiwan) have not been determined. In this study, we examined the effect of exposure to hypo (10‰)- and hyper (35‰)-osmotic salinity on hard clams raised at their natural salinity (20‰). The osmolality, [Na^+^], and [Cl^−^] of the hard clam hemolymph were changed in the same direction as the surrounding salinity. Further, the contents of total free amino acids including taurine in the gills and mantles were significantly upregulated in hard clam with increasing salinity. The gill Na^+^, K^+^-ATPase (NKA) activity, the important enzyme regulating cellular inorganic ions, was not affected by the changed salinity. Mantle NKA activity, however, was stimulated in the 35‰ SW treatment. The taurine transporter (TAUT) is related to the regulation of intracellular contents of taurine, the dominant osmolyte. Herein, a TAUT gene of hard clam was cloned and a TAUT antibody was derived for the immunoblotting. The TAUT mRNA expression of the mantle in hard clam was significantly stimulated in 35‰ SW, but protein expression was not modulated by the changed salinity. In gills of the hard clam with 10‰ SW, both TAUT mRNA and protein expressions were significantly stimulated, and it may reflect a feedback regulation from the decreased gills taurine content under long-term hypoosmotic acclimation. These findings suggest that TAUT expression is regulated differently in gills and mantles following exposure to alterations in environmental salinity. Taken together, this study used the physiological, biochemical and molecular approaches to simultaneously explore the osmoregulation in tissues of hard clam and may further help to understand the osmoregulation in bivalves.

## Introduction

Most marine mollusks are osmoconformers as the osmolality of the body fluids changes in the same direction as a change in environmental salinity (Mantel and Farmer, 1983). It would be expected that osmoconformation would result in osmotic stress to tissues of marine mollusks following changes in surrounding salinity. Systems that regulate both inorganic and organic osmolytes would be expected to be altered in response to changes in environmental salinity.

The transmembrane ion countertransporter, Na^+^, K^+^-ATPase (NKA) plays a major role in cellular ion-regulation. Although NKA activity was found in a range of tissues in the clam, *Rangia cuneat* (Saintsing and Towle, 1978), the highest activity was found in the mantle. NKA activity was also found to be highest in the mantle of the blue mussel, *Mytilus galloprovincialis*, while in another bivalve, *Scapharca inasquivalvis*, it was highest in the gills (Borgatti et al., 2003). These findings indicate that the mantles and gills NKA may play an important role in the response to alterations in environmental salinity.

In cellular osmoregulation, bivalves have the ability to mobilize organic osmolytes in addition to ion regulation as demonstrated in vertebrates (Somero and Bowlus, 1983). Long-term accumulation of inorganic ions causes damage to cells, and as such, inorganic ions are not conducive to long-term osmoregulation in cells (Yancey, 1994). In contrast, organic osmolytes are able to stabilize intracellular proteins without affecting cellular functions and suitable for the long-term accumulation in cells (Yancey, 1994; Burg et al., 2007). Hence, the modulation of intracellular organic osmolyte content is vital in multicellular organisms facing the changes in extracellular osmolality to maintain the cell volume (Yancey, 2005). In bivalves, the content of free amino acid (FAA) was higher in the intracellular fluid than vertebrates (Gilles, 1975, 1979). Previous studies indicated a large amount of FAA was accumulated in the intracellular fluid via the uptake of FAA from the hemolymph, *de novo* synthesis and/or the catabolism of cellular protein in bivalves upon hyperosmotic stress (Baginski and Pierce, 1975; Zurburg and DeZwaan, 1981; Deaton et al., 1985a; Ellis et al., 1985). Conversely, FAA was released to the hemolymph or extracellular fluid by modulating membrane and transporter properties upon hypoosmotic stress (Bartberger and Pierce, 1976; Baginski and Pierce, 1978; Bishop et al., 1981; Silva and Wright, 1992; Deaton and Pierce, 1994).

Taurine is the dominant FAA and high intracellular taurine concentrations existed during osmoregulation in marine invertebrates (Simpson et al., 1959; Lange, 1963; Allen and Garrett, 1971; Gilles, 1972; Bishop et al., 1983; Smith and Pierce, 1987; Yancey, 2005; Kube et al., 2006, 2007). In hyperosmotic environments, extracellular taurine was transported into the cell via the taurine transporter (TAUT) for the maintenance of osmolality (Wright and Secomb, 1984; Kwon and Handler, 1995; Burg et al., 1997; Bitoun and Tappaz, 2000a,b; Takeuchi et al., 2000, 2001). In reverse, taurine diffused out of the cells under hypoosmotic environments (Fugelli and Riersen, 1978; Fugelli and Rohrs, 1980). Bivalve TAUT was expressed in gills and mantles and mRNA expression changed with environment (Hosoi et al., 2005, 2007; Toyohara et al., 2005; Inoue et al., 2008). Similar to the vertebrate TAUT, the transport property of bivalve TAUT was related to external Na^+^ and Cl^−^ levels, and the high exogenous taurine levels caused the downregulation of mRNA expression and/or taurine uptake (Hosoi et al., 2005, 2007; Inoue et al., 2008).

The hard clam (*Meretrix lusoria*) is a marine inhabitant with extremely euryhaline behaviors throughout its life history. This species is an important aquaculture species in Taiwan and occurs naturally and is commercially cultured in about 20‰ seawater (SW) (Chen, 1976, 1990). However, the effects of changes in salinity on the cellular osmoregulatory mechanism of hard clam have not been investigated. Although previous studies had explored the response of TAUT mRNA expression in tissues of bivalves upon osmotic stress (Hosoi et al., 2005, 2007; Meng et al., 2013), the response of TAUT protein expression is not clear. In addition, there is no study simultaneously exploring the response of NKA and TAUT in bivalve with osmotic stress. It is required to explore whether these cellular osmoregulatory mechanisms work together in bivalves. In the present study, the hard calm, raised from 20‰ SW, were exposed to hypo- and hyperosmotic salinity (10 and 35‰) for 1 month and thereafter the osmolality and inorganic ions of hemolymph, as well as FAA contents, NKA activity, and TAUT expression (protein and mRNA) of gills and mantles of the hard clam were analyzed. By exploring these questions, it will help us to understand the mechanisms for the cellular osmoregulation of the hard clam.

## Materials and methods

### Animals and acclimation experiments

Hard clams, 30–50 mm and 16.5–18.5 g in body length and weight, respectively, were obtained from a local aquaculture farm [20‰ seawater (SW)]. To explore the effects of relative hypo- and hyper-osmotic stress on hard clam, 10, 20, and 35‰ SW solutions were used in the present study and were prepared from local tap water added with proper amounts of Instant Ocean synthetic sea salt (Aquarium Systems, USA). The hard clams were held in 20‰ SW for 15 days with a circulating system at 25°C under a 12:12-h light: dark photoperiod. In the salinity acclimation experiment, hard clams were transferred from 20‰ to 10, 20, and 35‰ SW for 1 month, and thereafter were sampled for further analyses. The protocol employed for the experimental hard clam was reviewed and approved by the Institutional Animal Care and Use Committee of the National Chung Hsing University (IACUC approval no. 96–48).

### Hemolymph analyses

The hemolymph of the hard clam was collected using the 23 G syringe. Samples were centrifuged at 14,000 g for 10 min at 4°C, and the supernatant was used for the analysis of osmolality, [Na^+^] and [Cl^−^]. Plasma osmolality was assessed with the WESCOR 5520 VAPRO Osmometer (ELI TechGroup, South Logan, UT, USA). [Na^+^] was measured with a Hitachi Z-8000 polarized Zeeman atomic absorption spectrophotometer (Tokyo, Japan). [Cl^−^] was evaluated using the ferricyanide method (Franson, 1985). Photometric analysis was conducted using a Hitachi U-2001 spectrophotometer (Tokyo, Japan).

### Content of FAA in the gills and mantles

The gills and mantles were freshly dissected from the hard clams, and then were freeze-dried (Osterode am Harz 3360, germany) for 10 h. The dried samples were mixed and incubated with 1 mL 2% SSA (sulphosalicylic acid, Sigma, St. Louis, MO, USA) at 4°C for 48 h. Thereafter, the incubated solution was centrifuged at 30,000 g, at 4°C for 30 min. After centrifugation, the supernatant was passed through a 0.22-μm filter (Millipore, Bedford, MA, USA). Finally, the filtered solution was analyzed by the Beckman 6300 High Performance Amino Acid Analyzer (Palo Alto, CA, USA).

### Specific activity of NKA

The NKA activities of the gills and mantles were determined according to the method of Hwang et al. (1989). The tissue was cut into pieces and then suspended in a mixture of homogenization medium (100 mM imidazole-HCl, 5 mM Na_2_EDTA, 200 mM sucrose, and 0.1% sodium deoxycholate, pH 7.6), and protease inhibitor (10 mg antipain, 5 mg leupeptin, and 50 mg benzamidine dissolved in 5 mL aprotinin) (vol/vol. 100:1). Homogenization was performed with a motorized Teflon pestle at 600 rpm for 30 s. The homogenate was then centrifuged at 13,000 g, at 4°C for 20 min. Next, the supernatant was used to determine protein concentrations and enzyme activities. Protein concentrations of the supernatant were determined by reagents from the Protein Assay Kit (Bio-Rad, Hercules, CA, USA), and bovine serum albumin (Sigma) was used as a standard. NKA activity was assayed by adding the supernatant to the reaction mixture (100 mM imidazole-HCl buffer, pH 7.6, 125 mM NaCl, 75 mM KCl, 7.5 mM MgCl_2_, and 5 mM Na_2_ATP). The reaction was run at 37°C for 30 min and then stopped by addition of 200 μL of ice-cold 30% trichloroacetic acid. The inorganic phosphate concentration was measured according to Peterson's method (Peterson, 1978). The enzyme activity of NKA was defined as the difference between the inorganic phosphate liberated in the presence and absence of 3.75 mM ouabain in the reaction mixture. Each sample was assayed in triplicate.

### Cloning of the taurine transporter (TAUT)

The mRNA was purified from total RNA of clam tissues with a commercial kit (Oligitex; Qiagen, Hilden, Germany). The cDNA for cloning and rapid amplification of cDNA ends (RACE) was made using a SuperScript III reverse transcriptase kit (Invitrogen, Carlsbad, CA, USA), and a Smart RACE cDNA amplification kit (Clontech, Mountain View, CA, USA) following the manufacturer's protocol. For PCR amplification, 3 μL of cDNA were used as a template in a 50 μL final reaction volume containing 0.25 mM dNTP, 2.5 units Ex-Taq polymerase (Takara, Shiga, Japan), and 0.2 μM of each primer. Primer sets obtained from Primer3 (http://frodo.wi.mit.edu/primer3/) are listed in Table 1. The obtained PCR products were subcloned into a pGEM-T Easy vector (Promega, Madison, WI, USA), and the amplicons were sequenced to confirm the PCR products. The specific primers of 5′ and 3′ RACE were designed from the partial sequences obtained from the PCR of the primer sets listed in Table 1. The program used for the RACE PCR followed the manufacturer's protocol, and RACE PCR products were also subcloned into the pGEM-T Easy vector and were sequenced.

**Table 1:** **Primer sets in the present study**.

**Name**	**Sequence (5′ to 3′)**
Degenerate-TAUT-F	TGGATHATHGTNGTNTTYTGYATHTGG
Degenerate-TAUT-R	RTARTCRAANARYTGRAANACRTACAT
TAUT-RACE-F	GGCAGATGCTGGGAACCAGGTGTT
TAUT-RACE-R	CCCTTCTACCCCAACAAACTGGCTGT
Q-TAUT-F	ATGTGCTCGAACTGGTTGCA
Q-TAUT-R	GAATTTCGTGCCAAGCTTTCA
Q-actin-F	TCCAGTTGTACGACCTGAAGCA
Q-actin-R	CAATCCAAAGGCCAACAGAGA

*TAUT, taurine transporter; F, forward primer; R, reverse primer; Q, qPCR*.

### RNA extraction and 1st cDNA synthesis

Each total RNA sample from the gill and mantle was extracted with RNA-Bee™ (Tel-Test, Friendwood, TX, USA) according to the manufacturer's instructions. The RNA pellet was dissolved in 50 μL DEPC-H_2_O and treated with the RNA clean-up protocol from the RNAspin Mini RNA isolation kit (GE Health Care, Piscataway, NJ, USA) according to the manufacturer's instructions to eliminate genomic DNA contamination. RNA integrity was verified by 0.8% agarose gel electrophoresis. Extracted RNA samples were stored at −80°C after isolation. First-strand cDNA was synthesized by reverse transcribing 5 μg of the total RNA using 1 μL of Oligo (dT) (0.5 μg μL^−1^) primer and 1 μL of PowerScript™ Reverse Transcriptase (Clontech) according to the manufacturer's instructions.

### Quantitative real-time PCR (qPCR)

The expression of TAUT mRNA was quantified with the Mini Opticon real-time PCR system (Bio-Rad). The primer sets arelisted in Table 1. The PCR reactions contained 8 μL of cDNA (1000 ×), 2 μL of either a 5-μM gene-specific primer mixture or a 5-μM β-actin primer mixture, and 10 μL of 2 × SYBR Green PCR MasterMix (Bio-Rad). The mRNA values were normalized using the expression of the β-actin mRNA from the same cDNA samples. The occurrence of secondary products and primer-dimers was inspected using melting curve analysis and electrophoresis to confirm that the amplification was specific. One identical control sample was used as the internal control among different groups. For each unknown sample, the comparative Ct method with the formula 2^∧^-([Ct _targetgene_, _n_- Ct_β-actin, n_] − [Ct _targetgene, c_ − Ct_β-actin, c_]) was used to obtain the corresponding TAUT and β-actin values, where Ct corresponded to the threshold cycle number.

### Specificity of TAUT antibody

The TAUT antibody was raised against the synthetic peptide ETTNSSSLDNLT according to the specific amino acid sequence from the hard clam TAUT (KU886300). The supernatant of gill homogenate (250 μg protein) was incubated in a vial with 1 μL of hard clam TAUT antibody at 4°Covernight. Next, a 20-μL slurry solution (50% protein A [Sigma], 1% BSA [Sigma]) was added into the vial and then incubated at 4°C for 1 day. Thereafter, the vial was centrifuged at 10,000 g for 1 min at 4°C and the supernatant was removed. The vial was washed with the IP buffer [140 mM NaCl (Merck, Darmstadt, Germany), 2 mM KCl (Merck), 10 mM Hepes, 5 mM EDTA (Merck), and protease inhibitor (as above description), pH 7.4] and then centrifuged. The washed IP buffer was removed from the vial and preserved for the immunoblotting analysis. For the elution, 30 μL Laemmli buffer (0.025 M Tris, 0.192 M Glycine, 0.1% SDS, and 5% β-mercaptoenthanol) was added to the vial, then the vial was heated at 65°C for 15 min. Finally, the vial was centrifuged at 10,000 g for 2 min at 4°C. The supernatant (eluted sample) from the vial was used for the immunoblotting analysis. The preserved and washed IP buffer (250 μg protein), eluted sample (20 μg protein), and the supernatant of gill homogenate (100 μg protein) were used for the SDS-PAGE gel electrophoresis and analysis of immunoblotting with the hard clam TAUT antibody. In the present study, the pre-immune serum was also used to replace the TAUT antibody in the immunoblotting test for antibody specificity.

### Immunoblotting of TAUT

The protein homogenate was prepared as described above, and was used for the immunoblotting. Aliquots containing 100 μg of the gill homogenates were heated at 100°C for 5 min and fractionated by electrophoresis on SDS-containing 10% polyacrylamide gels. Separated proteins were transferred from the unstained gels to PVDF (Millipore) using theMini Protean 3 tank transfer system (Bio-Rad). Blots were preincubated for 2 h in PBST (phosphate buffer saline with Tween 20) buffer (137 mM NaCl, 3 mM KCl, 10 mM Na_2_HPO_4_, and 2 mM KH_2_PO_4_, 0.2% (vol/vol) Tween 20, pH 7.4) containing 5% (wt/vol) nonfat dried milk to minimize non-specific binding. They were then incubated at 4°C with the clam TAUT antibody (TAUT), diluted in PBST (1:1500) overnight. After incubation with the TAUT antibody, the membrane was treated with goat anti-rabbit IgG AP-conjugated antibody (Chemicon, CA, USA) (1:2500 dilution) for 2 h. Blots were developed using NBT/BCIP kit (Sigma). Immunoblots were photographed and imported as TIFF files into the ID image analysis software package (MCID Analysis Evaluation7.0). Results were converted to numerical values in order to compare relative intensities of immunoreactive bands.

### Statistical analysis

Group data sets were confirmed to be normally distributed by the Anderson-Darling Normality Test (*p* < 0.05). The values are expressed as means ± SEM (standard error of the mean). The results in 10 and 35‰ SW group were compared with the control group (20‰ SW), respectively, via one-way ANOVA followed by Dunnett's test. *p* < 0.05 was considered statistically significant. *N*-value for the analyses of ions, osmolality and total amino acid is 8, and for the other analyses is 5.

## Results

### Salinity effects on osmolality and ion content of the hemolymph

**Figure 1:**
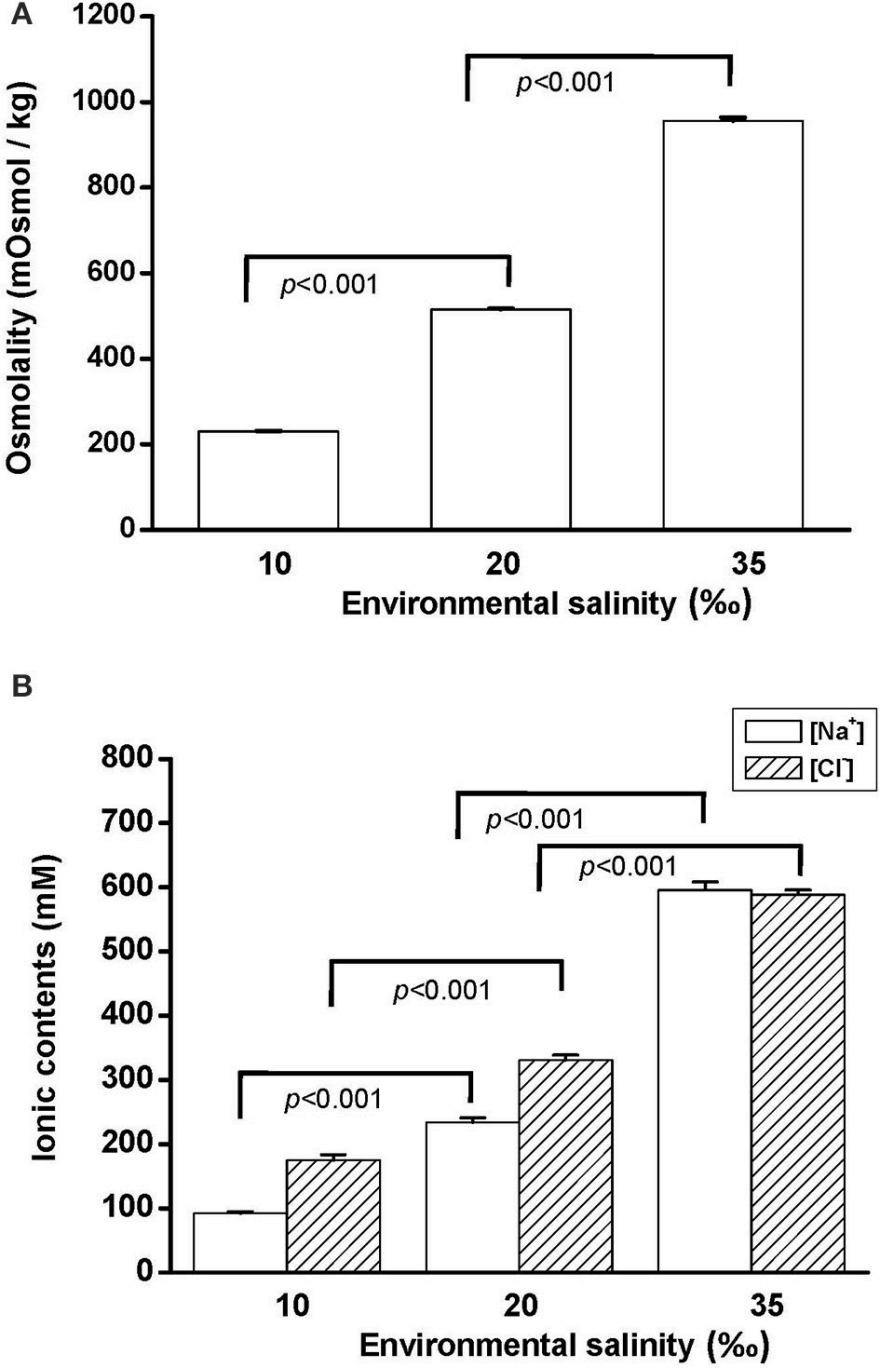
**Salinity effects on (A) osmolality and (B) [Na^+^] and [Cl^−^] of the hard clam hemolymph**. Statistical analysis was performed with one-way ANOVA followed by Dunnett's test. The 20‰ SW group was used as a control. *p* < 0.05 was considered statistically significant. Values are means ± SEM. (*N* = 8).

To explore the effect of hypo- and hyperosmotic salinity [10 and 35‰ seawater (SW)] on hard clam, the hemoplymph was collected from hard clam with 10, 20, and 35‰ SW treatment. Thereafter, the osmolality, [Na^+^], and [Cl^−^] of hemolymph were analyzed. The osmolality, [Na^+^], and [Cl^−^] of hemolymph in hard clam conformed with environmental salinity, and therefore the salinity-dependent upregulation in these physiological parameters was found (Figure 1).

### Salinity effects on NKA activity in the gill and mantle

**Figure 2:**
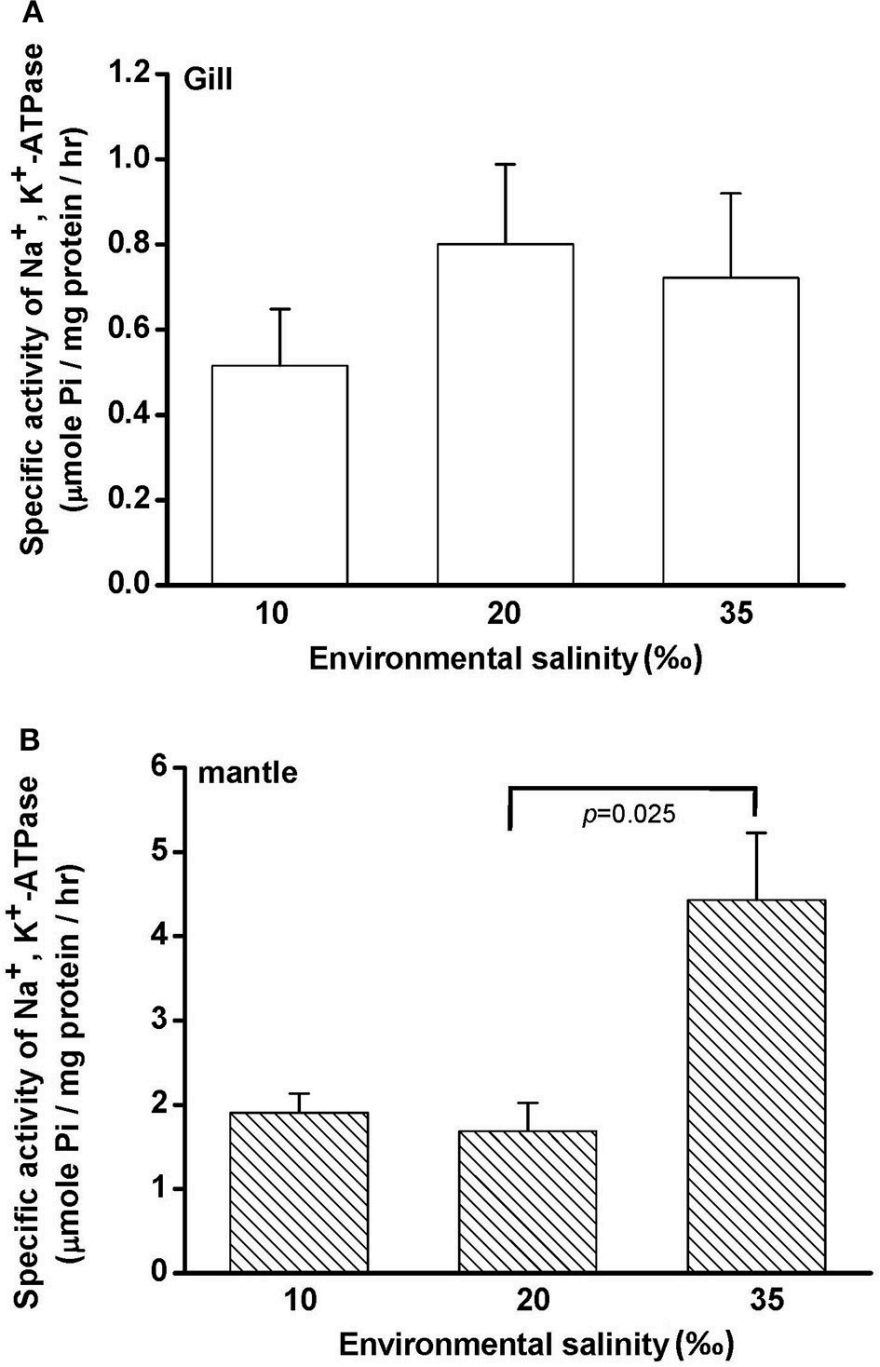
**Salinity effects on Na^+^, K^+^-ATPase activities in the gill (A) and mantle (B) of the hard clam**. Statistical analysis was performed with one-way ANOVA followed by Dunnett's test. The 20‰ SW group was used as a control. *p* < 0.05 was considered statistically significant. Values are means ± SEM. (*N* = 5).

Na^+^, K^+^-ATPase (NKA) activity is vital for cellular osmore-gulation by regulating the transport of ions. To explore the effect of hypo- and hyperosmotic salinity (10 and 35‰ SW) on NKA activity in hard clam, the gill and mantle of the hard calm with 10, 20, and 35‰ SW treatment were sampled for the assay of NKA activity. The hard clam with 20 ‰ SW treatment was used as a control group. Compared with control group, the NKA activity of gills in the hard clams was not changed by 10 and 35‰ SW treatment (Figure 2A). In the mantle, NKA activity was significantly stimulated by 35‰ SW treatment, but not changed by 10 ‰ SW treatment (Figure 2B).

### Salinity effects on FAA content in the gill and mantle

**Figure 3:**
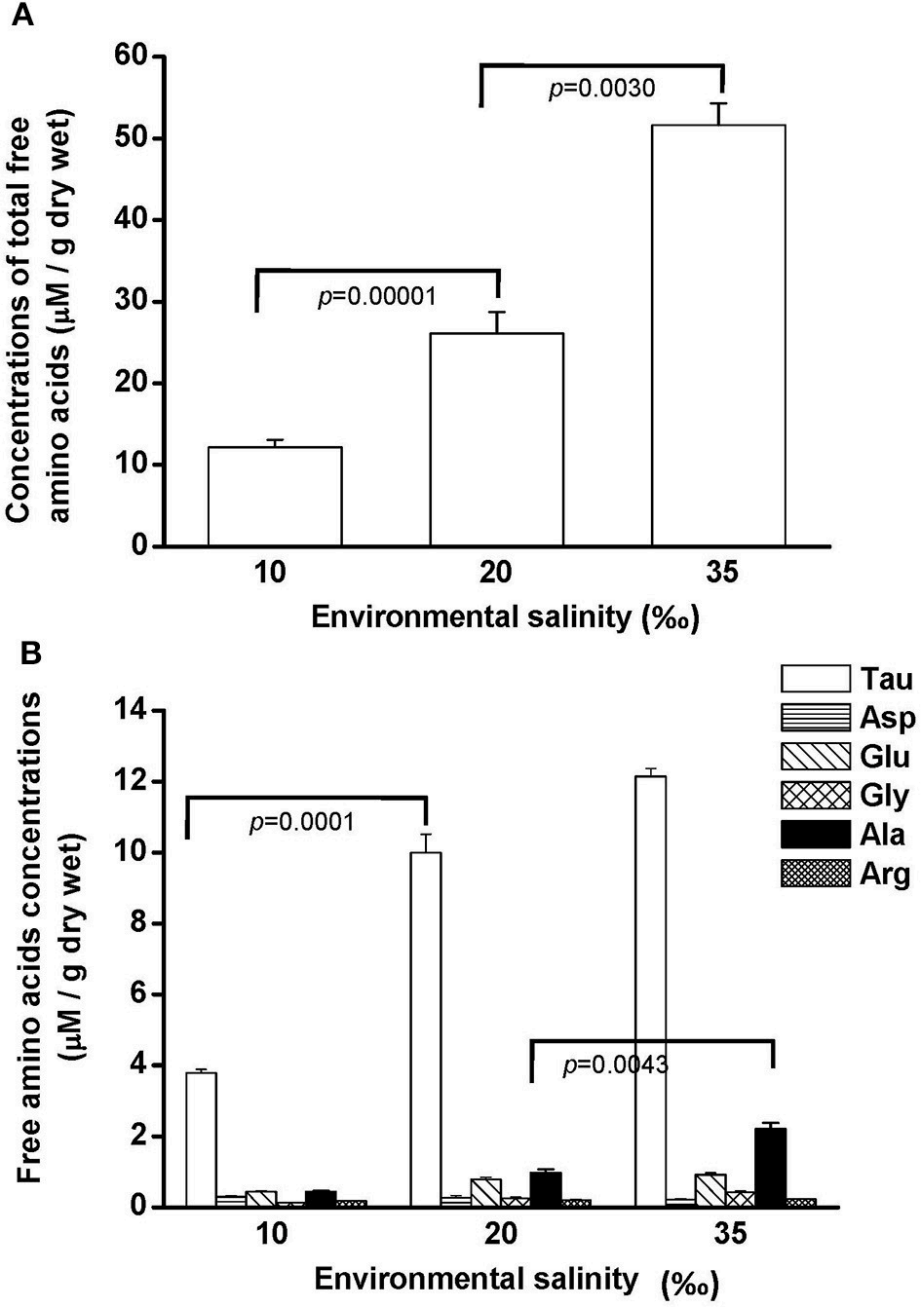
**Salinity effects on concentrations of total free amino acids (A) and different free amino acids (B) in gills of the hard clam**. Statistical analysis was performed with one-way ANOVA followed by Dunnett's test. The 20‰ SW group was used as a control. *p* < 0.05 was considered statistically significant. Values are means ± SEM. (*N* = 8). Tau, taurine; Asp, aspartate; Glu, glutamate; Gly, glycine; Ala, alanine; Arg, arginine.

**Figure 4:**
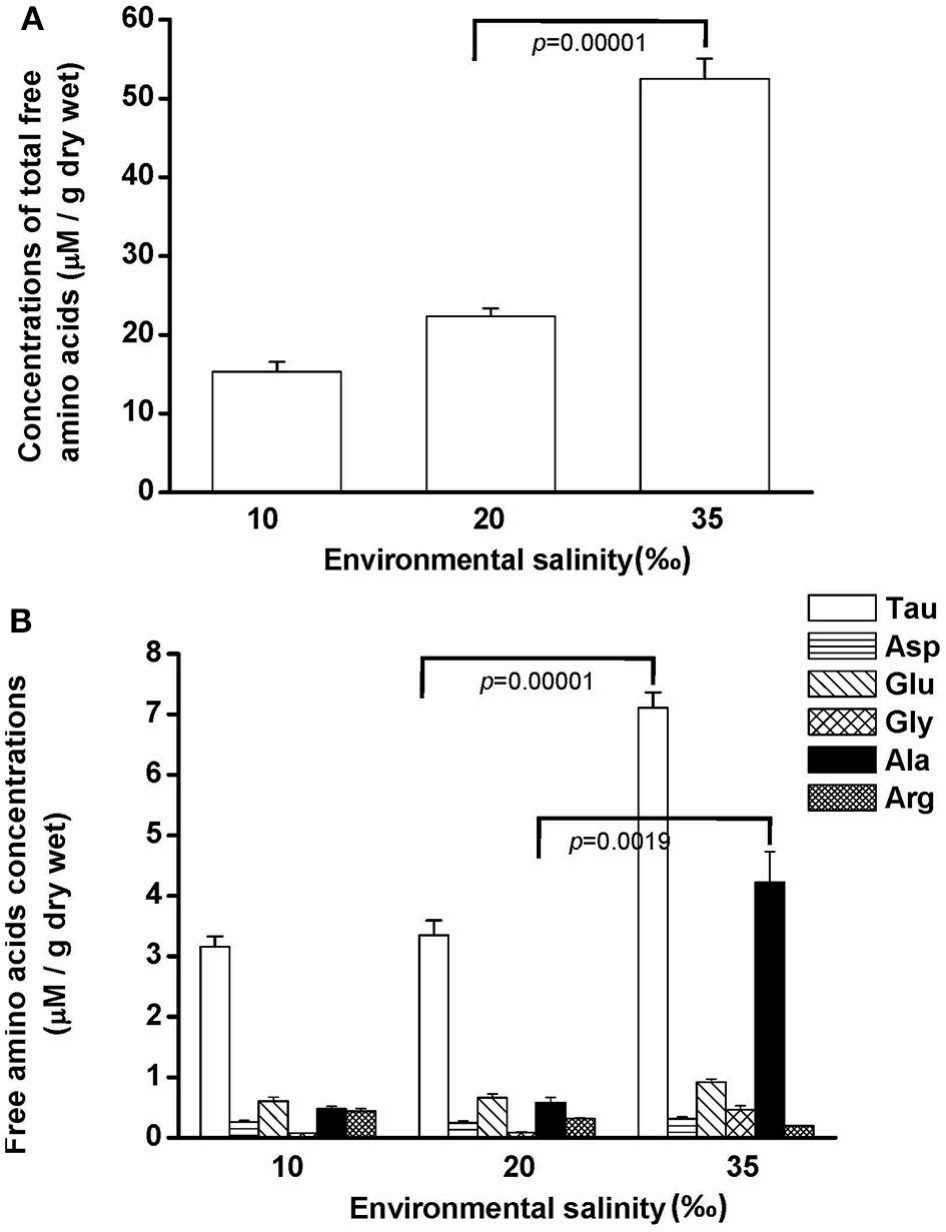
**Salinity effects on concentrations of total free amino acids (A) and different free amino acids (B) in mantles of the hard clam**. Statistical analysis was performed with one-way ANOVA followed by Dunnett's test. The 20‰ SW group was used a control. *p* < 0.05 was considered statistically significant. Asterisk indicates the significant difference (*p* < 0.05). Values are means ± SEM. (*N* = 8). Tau, taurine; Asp, aspartate; Glu, glutamate; Gly, glycine; Ala, alanine; Arg, arginine.

Free amino acids (FAAs) are important cellular osmolytes. To explore the effect of hypo- and hyperosmotic salinity (10 and 35‰ SW) on hard calm, the FAA component of the gill and mantle in hard clam with 10, 20 or 35‰ SW treatment was analyzed. Compared with control salinity, gill FAA contents of the clams were upregulated and downregulated in 35 and 10‰ SW treatment, respectively (Figure 3A). The mantle FAA content was also stimulated by 35‰ SW treatment, but not changed by 10‰ SW treatment (Figure 4A). In the compositions of FAA in gills and mantles, taurine had a dominant percentage (about 20–40%) (Figures 3B, 4B). The taurine contents of gills were significantly decreased and not altered and in the clams acclimated to 10 and 35‰ SW, respectively (Figure 3B). In addition, the taurine content of mantles was significantly increased in clams acclimated to 35‰, but not changed in clams acclimated to 10‰ (Figure 4B). Conversely, the alanine content of gills and mantles was significantly higher in the 35‰-acclimated hard clams than in control groups (Figures 3B, 4B). However, the alanine content of gills and mantles was not changed in clams acclimated to 10‰ (Figures 3B, 4B).

### cDNA cloning of TAUT and phylogenetic analysis

**Figure 5:**
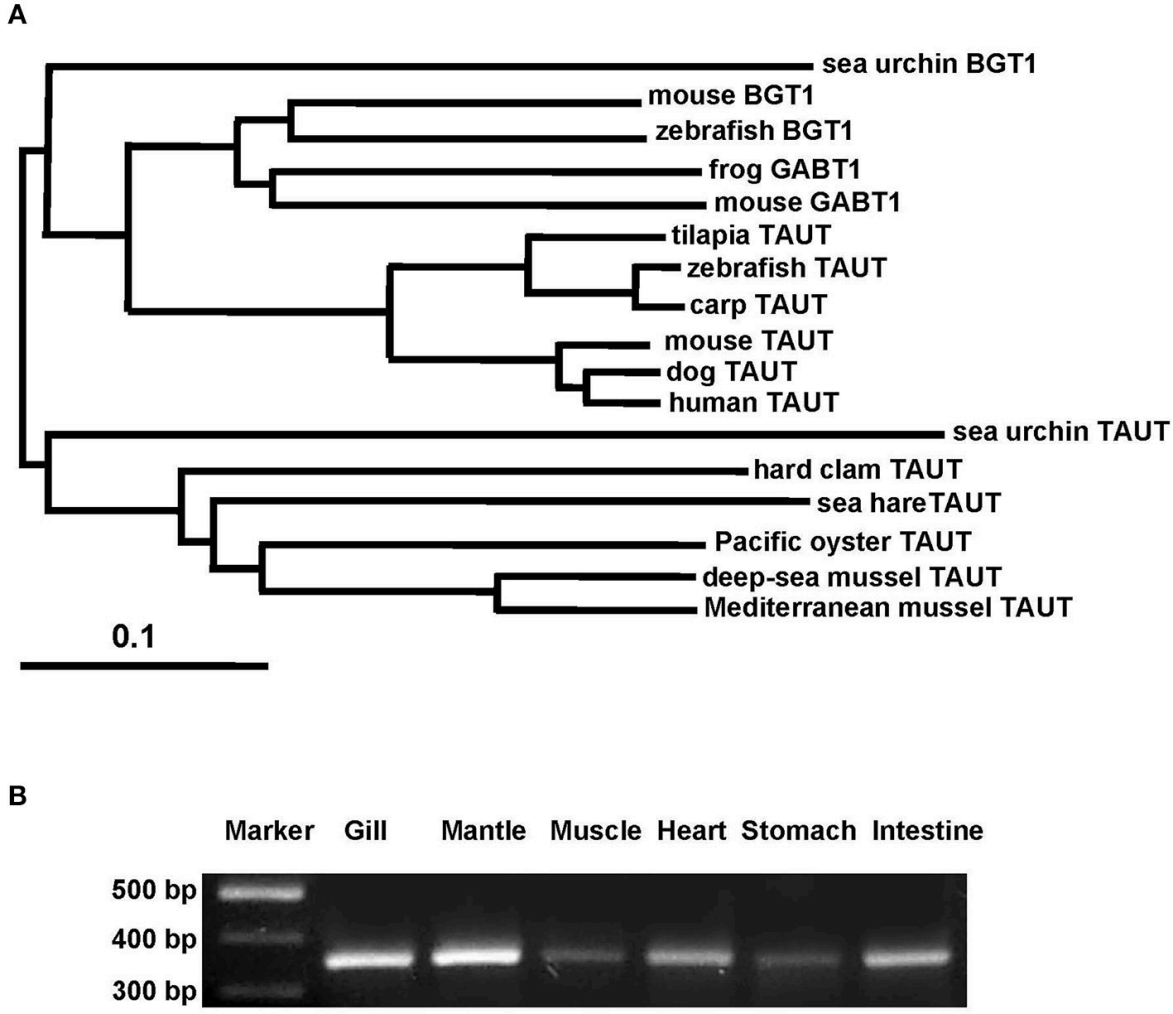
**Phylogenetic analysis of taurine transporter (TAUT) (A) and the tissue distribution of TAUT gene expression (B) in the hard clam. (A)** Full-length amino acid sequences of TAUT from different species were used to construct a phylogenetic tree by the neighbor-joiningmethod with 1000 bootstrap replicates. Percentages of bootstrapping branch corrections are shown beside the branches, and the ortholog relationships of clades are labeled on the right side to indicate different subtree regions. The GenBank accession nos. of the sequences used are as follows: Mediterranean blue mussel TAUT, BAD91313; deep-sea mussel TAUT, BAF95543; Pacific oyster TAUT, NP_001292278;sea hare TAUT, NP_001191394; sea urchin TAUT, XP_011676091carp TAUT, BAA89537; zebrafish TAUT, NP_001032750; tilapia TAUT, BAB18038; mouse TAUT, NP_033346; human TAUT, AAC50443; dog TAUT, NP_001003311; mouse BGT1, NP_598422; frog BGT1, XP_012815540;sea urchin BGT1, XP_785498; sea urchin GABT1, XP_784181; zebrafish GABT1, NP_001007363;mouse GABT1, NP_848818. **(B)** For the tissue distribution of TAUT gene expression, fragments were amplified by using gene-specific PCR primers. The TAUT is expressed in the gill, mantle, muscle, heart, stomach, and intestine. TAUT, taurine transporter; BGT1, betanine transporter; GABT1, GABA transporter.

Taurine is the most abundant FAA in the gill and mantle in hard clam and transported by taurine transporter (TAUT). To explore the effect of different salinities on TAUT expression in the gill and mantle of hard clam, a 1971-bp cDNA containing an open reading frame of 657 amino acid residues was cloned (KU886300) from the hard clams. The deduced peptide exhibited approximately 60% similarity to the TAUTs of the deep-sea mussel (*Bathymodiolus septemdierum*) and Mediterranean mussel (*Mytilus galloprovincialis*). The TMHMM program predicted 12 transmembrane structures, as observed in the TAUT of deep-sea and Mediterranean mussels (Hosoi et al., 2005; Inoue et al., 2008). According to the phylogenetic analysis (Figure 5A), the cloned TAUT sequences of the hard clamscould be assigned to the clade of molluscs TAUT.

### TAUT mRNA expression in different tissues of the hard clam

To explore the TAUT expression in different tissues in hard clam, TAUT mRNA expression of the hard clam was detected by RT-PCR in the gill, mantle, muscle, heart, stomach, and intestine (Figure 5B). Furthermore, expression levels were higher in the gill and mantle than in the other tissues examined.

### Specificity of the hard clam TAUT antibody

**Figure 6:**
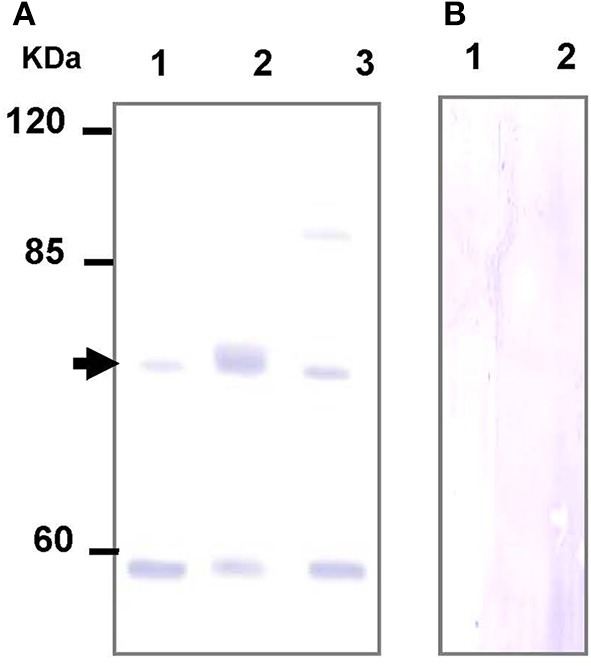
**Specificity analysis of the hard clam TAUT antibody**. The protein samples were separated by SDS-PAGE gel electrophoresis and performed immunoblotting with the **(A)** hard clam TAUT antibody or **(B)** pre-immune serum. **(A)** The predicted band at ~74 kDa could be found in the immunoblotting with the hard clam TAUT antibody. The arrow indicated the predicted immunoreactive band. Lane 1, 250 μg protein from the washed IP solution; Lane 2, 20 μg eluted protein from immunoprecipitation with TAUT antibody; Lane 3, 100 μg protein form the gill homogenate. **(B)** The pre-immune serum was used as the negative control, which revealed no immunoreactive band. Lane 1 and 2, 100 and 200 μg protein form the gill homogenate, respectively.

To further explore the role of TAUT in osmoregulation in the gill and mantle of hard calm, a specific antibody was derived to against hard clam TAUT protein. The molecular weight of the hard clam TAUT protein is ~74 kDa. In the present study, the predicted immunoreactive band at ~74 kDa was detected by immunoblotting with the hard clam TAUT antibody (Figure 6A). Moreover, the dominant band at ~74 kDa was found in the eluted sample of immunoprecipitation with the TAUT antibody (Figure 6A). In contrast, no immunoreactive band was detected by immunoblotting when the hard clam TAUT antibody was replaced with the pre-immune serum (Figure 6B). On the other hand, an unexpected band at ~58 kDa was also detected by the TAUT antibody (Figure 6A). Nevertheless, the immunoreactive band at ~58 kDa became weaker after the immunoprecipitation with the TAUT antibody (Figure 6A).

### Salinity effects on TAUT mRNA and protein expression in the gill

**Figure 7:**
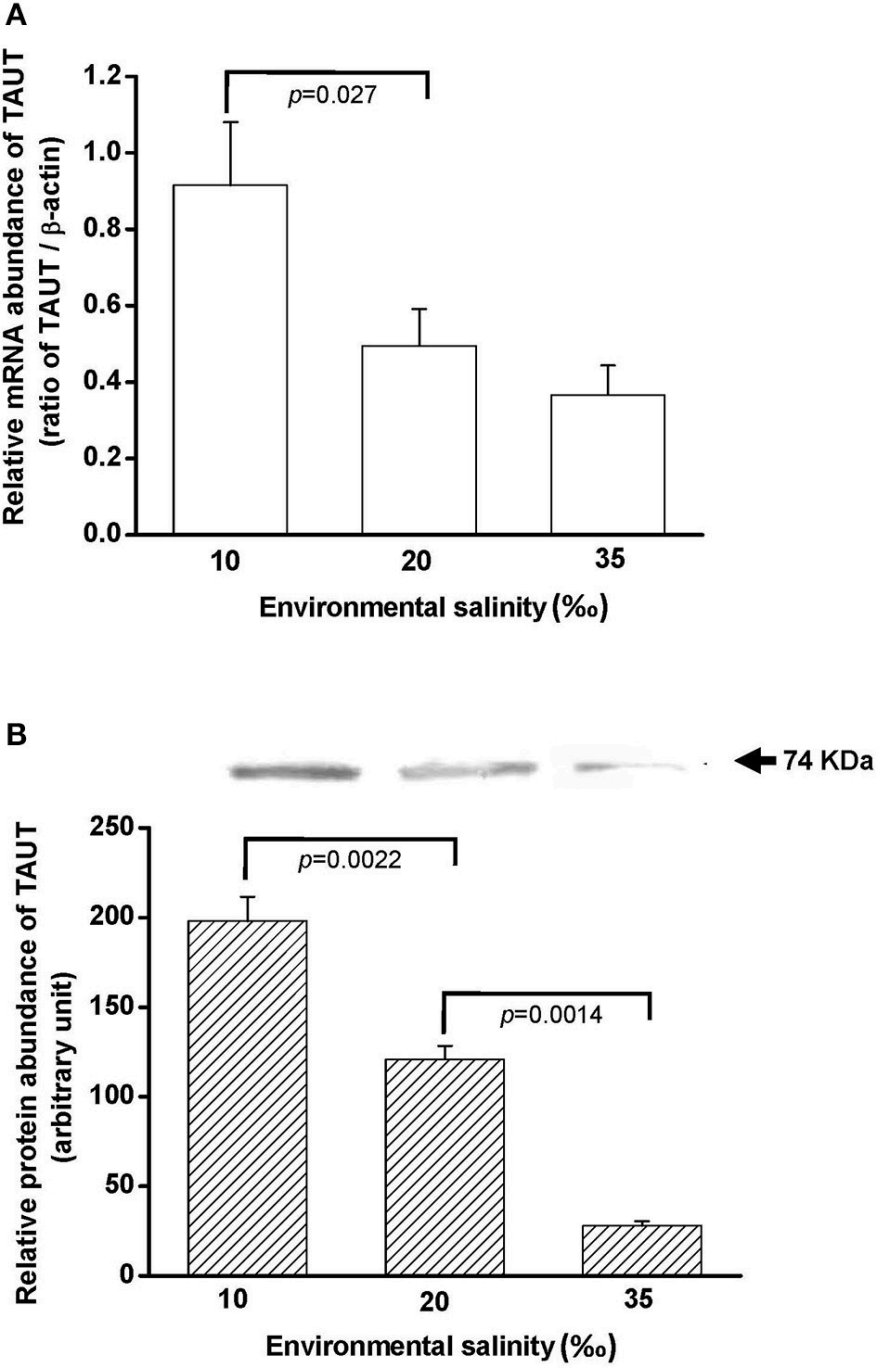
**Salinity effects on TAUT mRNA (A) and protein expression (B) in gills of the hard clam**. The representative immunoblot for the protein expression was shown in **(B)**. Statistical analysis was performed with one-way ANOVA followed by Dunnett's test. The 20‰ SW group was used as a control. *p* < 0.05 was considered statistically significant. Values are means ± SEM. (*N* = 5).

To explore the effect of hypo- and hyperosmotic salinity (10 and 35‰ SW) on TAUT expression in the gill of hard clam, the quantitative (Q) PCR and immunoblotting were performed to detect the mRNA and protein expression of TAUT. Compared with 20‰ SW treatment, the TAUT mRNA expression of the gills was significantly increased and not altered in the clam acclimated to 10 and 35‰, respectively (Figure 7).

### Salinity effects on TAUT mRNA and protein expression in the mantle

**Figure 8:**
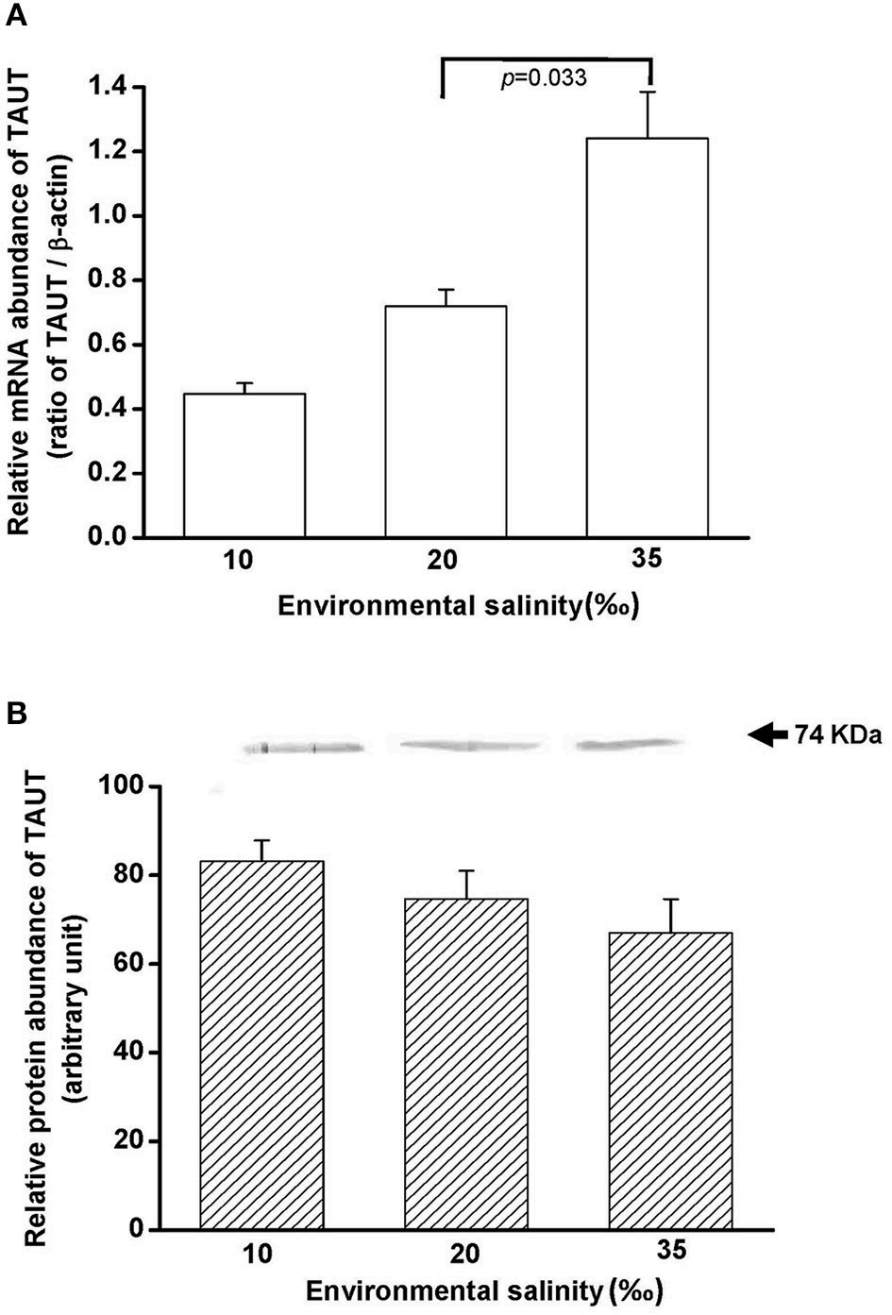
**Salinity effects on TAUT mRNA (A) and protein expression (B) in mantles of the hard clam**. The representative immunoblot for the protein expression was shown in **(B)**. Statistical analysis was performed with one-way ANOVA followed by Dunnett's test. The 20‰ SW group was used as a control. *p* < 0.05 was considered statistically significant. Values are means ± SEM. (*N* = 5).

To explore the effect of hypo- and hyperosmotic salinity (10 and 35‰ SW) on TAUT expression in the mantle of hard clam, the quantitative (Q) PCR and immunoblotting were performed to detect the mRNA and protein expression of TAUT. The TAUT mRNA expression of the mantle was significantly upregulated and not altered in the clams acclimated to 35 and 10‰, respectively (Figure 8A). However, protein expression of mantles was not altered in 10- and 35‰-acclimated hard clams (Figure 8B).

## Discussion

The findings of the present study show that the hard clam is an osmoconformer: hemolymph osmolality, [Na^+^] and [Cl^−^] changed in the direction of the change in salinity. Compared with the 20‰ group, the gill and mantle of clams acclimated to 10 and 35‰ seawater (SW) was immersed in hypo- and hyperosmotic environments. In the present study, we found that NKA activity was higher in the mantle than the gill (about 2-fold) in clams with 20‰ SW treatment. Moreover, NKA activity of the mantle was significantly stimulated in clams acclimated to 35‰ SW, whereas the NKA activity of the gill did not significantly change with ambient salinities. NKA actively transports Na^+^ out and K^+^ into cells and is the driving force for secondary ion transport of cells (Li and Langhans, 2015). Therefore, NKA activity may contribute to ion regulation in the mantle, but not in the gill during long-term hyperosmotic stress.

Generally, organic substances were thought to be the important osmolytes for osmoregulation of marine invertebrates (Yancey, 2005), and osmoregulation in the gill may depend mainly on organic osmolytes. The osmolytes, such as FAA are vital for cellular osmoregulation (Simpson et al., 1959; Zurburg and DeZwaan, 1981; Bishop et al., 1994; Burg, 1995). In the present study, the total FAA content in gills and mantles of the hard clam increased with elevated environmental osmolality. Hochachka and Somero (2002) stated that the contents of FAA occupied a considerable portion in the intracellular osmolytes, in addition to inorganic ions. Furthermore, the modulation in the content of organic osmolytes was an important strategy for hypo- and hyperosmotic adaptation in cells because of the low impact on cell physiology (Yancey, 2005; Burg et al., 2007). Hence, FAA contents in gills and mantles of the hard clam were modulated with ambient osmolality and reflected osmotic adaption. In the present study, the findings showed the FAA included taurine, aspartate, alanine, glutamate, glycine, and arginine. Among these measured FAA, taurine was the dominant. Indeed, Yancey (2005) stated that taurine was the dominant FAA in marine invertebrates. Several studies further revealed that taurine was the most copious osmolyte of bivalves (Gilles, 1972; Livingstone et al., 1979; Zurburg and DeZwaan, 1981; Deaton et al., 1985a,b; Hosoi et al., 2003; Toyohara and Hosoi, 2004; Kube et al., 2006, 2007). Taurine, a water-soluble osmolyte, can diffuse freely within cells and protect proteins, but did not interfere with intracellular physiology (Han et al., 2006). Several studies have reported that the contents of taurine in different tissues were relative higher in hyperosmotic acclimation in bivalves (Gilles, 1972; Livingstone et al., 1979; Hosoi et al., 2003). In the present study, taurine levels were significantly higher in the mantles but not altered in the gills of clam acclimated to 35‰ SW. In addition, other FAAs such as alanine contributed to the marked effect of 35‰ SW on FAA levels of gills and mantles since the relative ratio of taurine was lower at this salinity. According to the present results, at least taurine and/or alanine played a critical role in long-term osmotic adaptation in gills and mantles of the hard clam.

Taurine transporter (TAUT) is involved in the regulation of intracellular taurine content. In this study, taurine transporter (TAUT) of the hard clam was cloned. Phylogenetic analysis showed that this transporter belonged to the clade of mollusks TAUT. The activity and mRNA expression of TAUT were demonstrated to be inhibited by exogenous taurine supplementation, but were not modulated by glycine, alanine, or glutamic acid (Uchida et al., 1992; Jones et al., 1995; Han et al., 1997, 1998; Bitoun and Tappaz, 2000a,b; Takeuchi et al., 2000, 2001; Inoue et al., 2008). As others bivalves, e.g., deep-sea mussel, Mediterranean blue mussel, and Pacific giant oyster, the TAUT expression was detected in gills and mantles in the hard clam (Hosoi et al., 2005, 2007; Inoue et al., 2008). Hyperosmotic acclimation has been reported to induce the TAUT mRNA expression in mantles of the oyster and mussel (Hosoi et al., 2005, 2007). In the present study, exposure of hard clams to 35 ‰ SW also induced elevated mantle TAUT mRNA expression. We developed a specific antibody against the hard clam TAUT, and confirmed its specificity. The molecular weight of hard clam is about 74 kDa. In the present study, a predicted immunoreactive band at ~74 kDa was detected by the immunoblotting with the hard calm TAUT antibody, and the immunoprecipitation experiment further demonstrated the specificity of the hard clam TAUT antibody. Meanwhile, an unexpected immunoreactive band at ~58 kDa was detected by the TAUT antibody. Unlike the band at ~74 kDa, the band at ~58 kDa became weaker in the eluted sample of immunoprecipitation with the TAUT antibody. In addition, we did not find different expression of ~58 kDa among various salinity groups. Therefore, the band at ~58 kDa may not be the modified form of TAUT. Thereafter, the TAUT protein expression was analyzed by immunoblotting with the hard clam TAUT antibody. Unlike mRNA expression, the protein expression of mantle TAUT was not altered in calms acclimated to 35‰ SW. However, the taurine contents of mantles of the 35‰ SW-acclimated hard clams were still significantly higher. The bivalve TAUT activity is stimulated by the increased extracellular Na^+^ and Cl^−^ level (Hosoi et al., 2005, 2007; Inoue et al., 2008). Therefore, the higher extracellular Na^+^ and Cl^−^ level in 35‰ SW was suggested to modulate the mantle TAUT activity and resulted in increased taurine content. In contrast to mantles, the TAUT protein and mRNA expression in gills were upregulated in clams acclimated to 10‰ SW, rather than to 35‰ SW. The mRNA expression of TAUT in gills was also dominantly upregulated in the mussel and oyster acclimated to hypoosmotic environments (Hosoi et al., 2005, 2007). The upregulated TAUT mRNA expression in the giant Pacific oyster upon hypoosmotic stress was suggested to compensate the decreased taurine content following a dominant decline in the internal osmolality (Hosoi et al., 2007). In the present study, we found the taurine content of gills was significantly downregulated in 10‰ SW. Thus, the significantly higher gill TAUT expression found in the clam of the 10‰ group may also reflect the decreased in taurine content in the gill, which caused a secondary feedback of TAUT expression. Herein, we found that the contents of alanine were upregulated in gills and mantles of the clam acclimated to 35‰ SW. Previous studies reported that the intracellular contents of alanine in the marine bivalve, *Macoma balthica*, were positively associated with habitat salinities (Kube et al., 2006, 2007). In the Pacific oyster, the mRNA expression of glutamate decarboxylase (GAD), the final step enzyme to synthesize alanine, was significantly stimulated under hyperosmotic salinity (Meng et al., 2013). Therefore, the GAD expression may be also stimulated in hard clam upon hyperosmotic condition and contributed to increased alanine in the present study.

In conclusion, this study illustrated the osmoregulatory mechanisms of the hard clam by elucidating the osmolality of hemolymph, as well as the FAA contents, NKA activity, and TAUT mRNA/protein expression of gills and mantles under hypo- and hyperosmotic salinities. As an osmoconformer, the osmolality of hemolymph in hard clam conformed to external osmolality. The FAA contents in gills and mantles were stimulated by hyperosmotic salinity. The changes were suggested to cope with external osmotic challenge. However, the NKA activity and TAUT mRNA/protein expression between gills and mantles under the external osmotic stress was regulated differently. The present study demonstrates that there are tissue-specific responses in osmoregulation due to differences between the gills and mantles.

## Author contributions

C-HL, P-LY, and T-HL conceived and designed the research. C-HL and P-LY carried out the experiments. C-HL, P-LY, and T-HL wrote the manuscript. T-HL supervised the project. All authors approved the manuscript for publication.

### Conflict of interest statement

The authors declare that the research was conducted in the absence of any commercial or financial relationships that could be construed as a potential conflict of interest.
